# An Autophotographic–Phenomenological Investigation of British Transmen’s Psychological Well-Being

**DOI:** 10.3390/healthcare14030389

**Published:** 2026-02-03

**Authors:** Harry Cosford, Iain Richard Williamson

**Affiliations:** Psychology, Faculty of Health and Life Sciences, School of Applied Social Sciences, De Montfort University, Leicester LE1 9BH, UK

**Keywords:** gender-expansive, transmen, healthcare, well-being, visual methods

## Abstract

**Background/Objectives**: British trans and gender-expansive individuals face stigma and consistently experience lengthy waits for gender-enhancing interventions. Researchers are using a range of qualitative methodologies to give this marginalised community a voice. In this study the focus is on the lived experiences of British transgender men seeking medical intervention around their gender identity. The aim was to explore how psychological well-being for this group of transmen was both threatened and supported. **Methods**: Online semi-structured interviews using auto-photography were conducted with eleven transmen aged between 18 and 68 years. Both verbal and visual data were analysed together using interpretive phenomenological analysis. **Analysis**: Three themes focus on challenges and supportive strategies utilised by participants both before their decision to transition and after. All participants expressed significant mental health difficulties before commencing their transition, typically originating from childhood and continuing until they gained access to gender-affirming medical care. Their transition journeys damaged their well-being through resistance and rejection from families and communities, and difficulties navigating healthcare systems. A series of resources which significantly enhanced well-being were also reported. Unconditional acceptance and belonging found within and beyond the trans community, connecting with nature and ultimately progressing with gender-affirming healthcare were key elements in protecting and promoting well-being. **Conclusions**: The toll on the well-being of trans and other gender-expansive individuals is considerable and recent changes in UK law have exacerbated the hostile environment faced by TGE individuals. Community-based allyship and access to affirmative professional psychological support at all stages is vital.

## 1. Introduction

Qualitative scholarship on individuals who identify as non-cisgender is accelerating rapidly. Trans and gender-expansive (TGE) individuals and communities are becoming more visible and mobilised in many countries but also face very significant discrimination across all parts of the socio-ecological systems they inhabit, which typically threatens their rights and well-being on a quotidian basis [1]. In an often hostile social environment, the mental health of many TGE individuals is an area of current and considerable concern. This article therefore focuses on experiences of transmen in the United Kingdom and especially how positive and negative experiences of healthcare affect their psychological well-being. To contextualise the study which focuses on the well-being of British transmen and their healthcare experiences specifically, background material is provided on the size of the British TGE community, the mental health burden experienced by this community, the contemporary socio-political climate of TGE rights in the UK and an explanation of how medical transition processes operate.

Estimates suggest that around 1% of the population of the United Kingdom (UK) identify as transgender or gender-expansive (TGE) [2]. TGE identities have traditionally been pathologized, marginalised and medicalised and individuals identifying as TGE have often faced various distal and proximal threats to well-being, including inter-personal discrimination and violence, structural stigma and negative representations in public discourse [3,4,5]. Cisnormativity and transphobia are daily features of almost all TGE individuals’ daily lives [6].

Given this environment, it is arguably unsurprising that research consistently shows significantly high rates of self-harm, suicide and suicide attempts in the TGE community [4]. One British survey of TGE adults reported a lifetime suicidal ideation of 84% and suicide attempts of 48% [7]. A recent synthesis suggested that for transmen, anxiety, depression and PTSD [8,9] are consistently well above both population-levels. According to the Gender Minority Stress Model (GMSM), there are many external stressors like rejection, discrimination, exclusion, lack of support, victimisation, misgendering and prejudice which lead to a rise in internal minority stressors like nondisclosure (sometimes known as ‘passing’ or ‘living in stealth’), internalised stigma and negative expectations of future situations and therefore result in negative outcomes such as mental health difficulties and suicidal ideation [4]. The GMSM also demonstrates how the cumulative effects of such micro-aggressions like dead-naming and deliberate gender pronoun misuse negatively impact quality of life and mental health [10].

TGE communities are both diverse and evolving, including both individuals who wish to transition (medically and/or socially) from their biological sex to a male or female gender identity as well as individuals who favour a non-binary or more fluid gender identity, often exemplified by the use of ‘they’ or alternating pronouns [11]. In supporting gender-expansive individuals and organisations, scholars, activists and practitioners are increasingly aiming to understand and address some of the psychosocial and structural threats to well-being faced by the gender-expansive community in their praxis [12]. Population heterogeneity is culturally enriching and can allow for shared activism and mobilisation, but challenges faced by different TGE sub-communities can and do vary. Therefore, further research, especially within sub-communities of the gender-expansive community, is required to inform and enhance advocacy and practice [13]. Transmen represent one group of TGE individuals who are often under-represented in both research and public discourses around trans rights and well-being [14,15].

Whilst recent years have seen some increases in activism and allyship in many countries, the rights of transgender individuals remain both limited and highly contested, especially in areas such as healthcare, education and sports where in some contexts new legislation is being passed to inhibit the rights of TGE people further [16,17].

Acceptable and affirmative language to represent non-cisgender individuals is complex and contested [18]. While the term ‘transition’ refers to the route TGE people embark on to change their expression and presentation to parallel with their gender-expansive identity, each ‘transition’ journey for each gender-expansive individual is unique [19] and in many ways the concept of transitioning is problematic. Whilst many TGE individuals wish to transition medically through gender-affirming treatment, for others their journey involves social transitions such as changing their name and self-presentation [10], whilst others elect to represent themselves in more gender-fluid and/or non-binary ways that are less commonly related to legal protections [20].

In the UK, in line with the World Professional Association for Transgender Health [21], transgender people in the UK must have a psychiatric assessment for ‘gender incongruence’ and display a consistent, stable gender identity over a specific amount of time before they can access gender-affirming medical treatment through British healthcare services (the free-to-all National Health Service) [22].

Those who chose the medical transition route of pursuing treatment for Gender Dysphoria in the UK are usually referred by a medical profession to one of the UK’s twelve Gender Identity Clinics (GICs). In recent years referrals to GICs in the UK have increased significantly, with referral rates rising proportionately more for transmen than transwomen [23]. Most GICs report unprecedented increases in the amount of people (particularly young people) seeking support with their gender identity [24]. For example, Tavistock, one of the UKs largest GICs, reported that their waiting list has increased from 97 in 2009 to 12,296 in 2023 [25]. Therefore, despite NHS guidelines stating that there should be a maximum of 18 weeks wait time between a GP referral and a first appointment with a GIC [26], TGE people are facing extensive waiting times to access gender-affirming medical care, with waiting times of between 18–82 months for an initial appointment with a GIC [23]. In addition there appears to be very little support, information and integration of care for those in a transitioning individual’s familial or community networks [27].

Whilst some clinicians are empathic and compassionate, research suggests that TGE individuals regularly report discrimination, stigma and explicitly negative attitudes from many healthcare professionals [28,29]. Studies from around the world suggest that many healthcare professionals lack knowledge about transgender healthcare and identities, which often results in transgender patients reporting the experience of abusive treatment, discriminatory attitudes, refusal of access to appropriate healthcare, misgendering, inappropriate questioning and receiving insensitive, prejudicial and negative treatment from healthcare professionals [30,31]. Additionally, research demonstrates that medical professionals in the UK rarely receive training on TGE healthcare, with some studies suggesting that within the medical curriculum, TGE individuals are either inadequately covered or not discussed at all [32].

Identity affirmation is key for a positive self-identity [3]. Research suggests that supportive, reliable and well-functioning social relationships are vital as they can serve as protective factors which improve TGE people’s well-being and mitigate the negative effects of minority stress on their psychological well-being. Studies suggest that TGE people who have the support of close friends and family, particularly during key stages in their transition process, have higher self-esteem, enhanced self-image and greater resilience in comparison to those rejected by their family and friends [33,34]. Additionally, support from LGBTQ networks has been found to have positive effects on transgender people’s mental health, with studies suggesting that stigmatisation can be less challenging when aligned with other individuals whose identities also question sexual or gender norms [35], although some TGE individuals have cited experiences of exclusion and transphobia from LGB communities [10].

Understandably, especially given reliably reported poor(er) health outcomes, much of the theorising and research around TGE individuals and communities in psychology has examined contributing factors to poorer health and well-being. In highlighting these negative effects, there is a danger that the narrative, although sometimes voiced from a position of concern and compassion, further disempowers TGE individuals and implies that stigma is intractable and internalized rather than managed and resisted. Increasingly we are seeing the emergence of a body of research that focuses on resilience and other resources (both intrapersonal and relational) that TGE people develop and utilise in the promotion and maintenance of well-being. Such ‘assets’ are often referred to as ‘salutogenic’ following a theoretical model offered by Antonovsky [36]. Taking a more holistic approach was important to both researchers and helps inform potential interventions more effectively than relying on risk and deficit models of distress. Therefore, through the elicitation and utilisation of integrated verbal and visual accounts, the researchers aimed to explore the narratives of British TGE individuals around both what helps and harms well-being.

Activism and theorisation around TGE individuals and communities has been gaining ground internationally [3,37], yet empirical research which is not primarily deficit-focused remains limited in a British context [38,39,40,41] and there remains very limited research specifically on transmen despite their increasing prevalence and visibility [42,43]. This study therefore aimed to explore the following research questions: What threatens the psychological well-being of British transmen and How do British transmen understand, protect and cultivate their well-being while navigating life as a TGE person?

The study had several objectives:

1. To collect in-depth data from a sample of British-based transmen to understand how they experience and aim to preserve psychological well-being.
2. To integrate auto-photography and interview methods to allow participants two modes of account-giving.
3. To elicit and interrogate experiences of transmen’s progress through British gender-affirming treatment and how these affect identity and well-being.

## 2. Materials and Methods

### 2.1. Theoretical Design

Data for this study were collected through interviews and participant-authored photography and analysed using Interpretative Phenomenological Analysis (IPA). With its theoretical roots of idiography, phenomenology and hermeneutics, IPA is centred on the comprehensive examination of a person’s lived experiences, how people make sense of their experiences and the meaning that they attach to such experiences [44]. However, as Smith [44] argues, such experiences cannot be simply plucked from the minds of participants and thus require examination and interpretation by a researcher, which links to IPA’s hermeneutic perspective [44]. By drawing on hermeneutics, an IPA typically blends descriptive and interpretative processes [45]. IPA is also idiographic as it involves analysing each participant’s experience in detail while searching for similarities across all the participants’ experiences and retaining some of the individual nuances of participants as the data are presented and discussed [44].

Semi-structured interviews are considered to be the prototypical method of collecting data in IPA studies, allowing for the acknowledgement that the participants are the ‘experiential experts’ in this area and interviews give them the chance to tell their story to the researcher in detail [45]. The intensive nature of analysing each case in such detail means that IPA studies are best conducted with small sample sizes [44]. Furthermore, research demonstrates that incorporating a photo-elicitation component allows participants to use their own photographs to reflect on real-life experiences, giving them the opportunity to share things that have meaning and importance to them as well as capturing meaningful moments of everyday experience ‘in real time’ [46]. The photographic element arguably enhances both the participant-led nature of narrative development and enhances the idiographic component of the phenomenological process [46].

### 2.2. Participants

Eleven British transmen took part in this study (See Table 1). They were aged between 19 and 68 years old and were living in various locations throughout the UK. Participants were asked to choose a pseudonym and self-labelled their TGE identity. Participants were required to be over the age of 18, living in the UK, identifying as transmen and needed to be receiving or on NHS waiting lists for gender-affirming medical interventions. This final criterion helped maintain homogeneity in the sample and allowed us to explore experiences in a healthcare context. As can be seen, various terms were used by participants to self-label, although all responded to a recruitment flyer seeking transmen so (whilst recognising the limitations of our approach) we have used this term throughout the paper for coherence and continuity.

### 2.3. Materials

A flyer was used to recruit participants who then received a participant information sheet, consent form and debrief sheet. Data were collected via a semi-structured interview schedule and a photo activity instruction sheet. We have included these as Appendix A and Appendix B for transparency. Participants also completed a short demographics questionnaire.

### 2.4. Procedure

After ethical approval was granted by the host University (reference: 540320), the information sheet, photo activity instructions, demographics forms and interview schedule were shared with two support group leaders prior to participant recruitment. This allowed for the support group organisers to provide feedback on all participant-facing materials and ensured that they were appropriate. The support group organisers then advertised the study to their groups and interested members emailed the researchers, who provided the participant information sheet, demographics and consent forms. Once the participants filled out these forms, the researcher then emailed the participants asking for around five photos representing their well-being, attaching the photo activity instruction sheet. Online interviews were then arranged.

All online interviews were conducted and transcribed using Microsoft Teams software between April and July 2023. Eight of eleven participants provided photographs, discussion of which bookended the interviews. Interviews lasted between 23 and 78 min long and were fully anonymised using participant-chosen pseudonyms.

### 2.5. Analytical Strategy

Data were analysed using IPA methodology following the recommendations of Smith and colleagues [47]. After each interview was transcribed, it was immediately and fully anonymised. Photographs were embedded into the transcripts at the point at which they were discussed and redacted if including any identifiable components. The composite verbal and visual data transcripts were subsequently read and re-read multiple times, allowing the first author to become familiar with the data. By analysing each transcript in turn, this allowed an idiographic perspective to be maintained. Notes were then made in the margins of each transcript regrading recurring motifs, points of interest and language used. These notes were then used to create ‘experimental statements’ which allowed for important features of the participants’ stories, including photographs, to be captured [47]. Mind maps were then created for each transcript to identify individual comments and associated images in the search for emergent group-level themes. All interviews were searched for commonalities and differences, which allowed for the creation of major themes. As IPA studies use small sample sizes, this allowed the individual transcripts to be analysed and interpreted in detail, while identifying key connections and experiential themes which emerged between the transcripts. These were then sorted into group experiential themes and quotations and images were used to ensure the idiographic element of each participant’s account was retained. Photographs were not individually coded for analysis or examined from a technical or semiotic standpoint, but rather seen primarily as prompts to elicit and provide reinforcement to verbal accounts. At this point the second author worked through the transcripts and tables, reviewing and refining the analysis in discussion with the first author. With regard to the images, technical and structural properties of the photos were typically not analysed in detail as part of this study; however, observations have been included where relevant to enhance the analysis. For the three participants who preferred not to take photographs, analysis was simply carried out on the verbal data. As the conceptual and thematic material covered in photographs was mostly very similar, there were not major differences in the content of data derived solely from interviews compared to combined verbal and visual accounts, although typically interviews without discussion of photos were shorter.

## 3. Analysis

**Figure 1:**
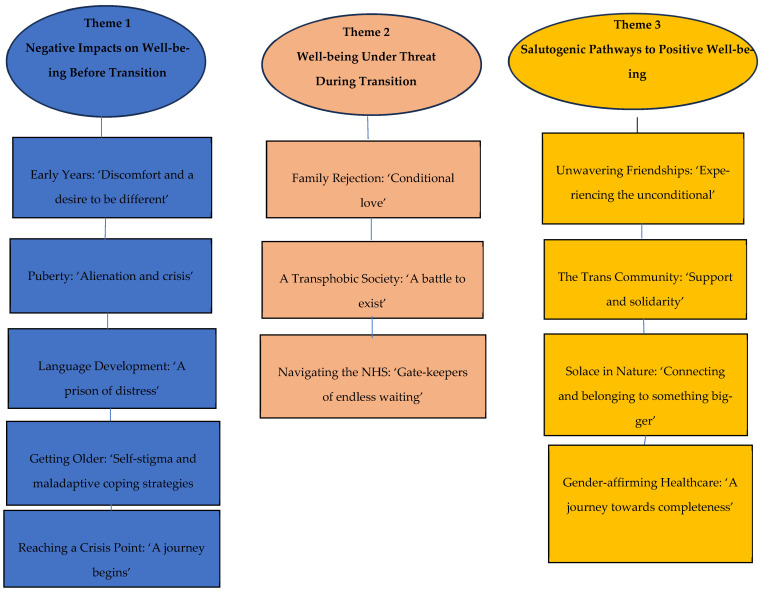
Schematic map of themes.

The analytic process generated three major themes which were particularly pertinent to well-being: (1) Negative impacts on well-being before transition (2). Well-being under threat during transition (3). Salutogenic pathways to positive well-being (See Figure 1).

Participants framed their accounts in a broadly chronological manner. Each theme comprises several subthemes. These are outlined, explained and discussed and illustrated by images and quotations from the participants.

### 3.1. Negative Impacts on Well-Being Before Transition

This theme captures the distress and discomfort that the participants experienced from a young age, through to starting their transition journey. This theme is made up of five sub-themes: early years, puberty, language development, getting older and reaching a crisis point.

Early Years: ‘Discomfort and a desire to be different’

Most of the participants shared that their feelings of being different began at a young age. While they could not comprehend what their feelings meant, they characterised these feelings in a negative way:

*“I was about 4 years old when I knew I was different… I felt so uncomfortable even at such a young age”*

(Lucas).

*“… 3 or 4 years old… when I was aware that there was something not right in my life”*

(Martin).

While the participants could not understand this feeling of being different, partly due to a lack of information about transgender people, they understood their desire to be a boy very clearly:

*“There was no word transgender in circulation at that time… I just knew that more than anything I wanted to be a boy”*

(Ryan).

*“… When I was 7 or 8 years old… thinking and really knowing… I was a boy… just assuming I was going to grow up to be a boy”*

(Richard).

These responses suggest an innate sense of gender. Being aware of their gender incongruence at this early age resulted in them experiencing poor well-being and subsequent discomfort in their bodies. By describing themselves in ways that suggest something was wrong with them, this demonstrates how the participants began negatively evaluating themselves from a young age as something that needed to be fixed. Ryan’s comment, for example, illustrates how despite a lack of understanding of what being transgender meant, the desire to be a boy was so clear that he assumed he would grow into being a boy. Such comments provide support for transgender identity development theories which explain that the early years for transgender people are characterised by feelings of being different, confusion and attempts to gather information about their identity which does not mirror their authentic self, instead developing unnoticed by society [48,49,50].

Puberty: ‘Alienation and crisis’

Participants went on to describe how their struggles with gender incongruence were magnified during puberty, a stage that became particularly negative for their well-being:

*“I kind of first went into that breakdown when I was 11–13… I started to get suicidal thoughts… and thoughts of self-harm”*

(Richard).

*“… When puberty hit… reality came crashing in and… I became very depressed… I was admitted to a psychiatric hospital with suicidal depression at 19… and prescribed electro-convulsive therapy”*

(Ryan).

For most of the participants school was clearly their first experience of rejection, which resulted in the internalising of feelings of rejection. Alan described difficulties fitting in with and relating to his peers:

*“When you are a young teenager… you really desperately want to fit in with your peers… when there’s something that’s like a barrier there to be able to relate to them, to relate to yourself… it’s very isolating”*

(Alan).

This suggests that puberty was a particularly distressing age for the participants where their well-being declined significantly and thoughts of suicide, depression and self-harm began. To fit in with their peers they concealed their identities, but this brought about feelings of isolation and being unable to relate to others. Thus, along with an absence of a sense of belonging to their peers, puberty presumably started to give them a sense of the development of an adult body that they do not want, which therefore negatively affected their well-being. These findings concur with research that show that minority stress and gender dysphoria are heightened in adolescence and that an individual’s thoughts, emotions and behaviours are regulated by external demands, driven by a longing to by accepted by others [7,51]. Subsequently the suppression of identity has amplificatory impacts on emotional and psychological distress, resulting in transgender adolescents being vulnerable to suicidal thoughts, self-harming behaviours and depression [52,53].

Language Development: ‘A prison of distress’

An advantage that age gave the participants was the development of language to describe clearly what it was like being a treatment-seeking transgender man before having access to gender-affirming medical care:

*“I felt like my body was a prison… which would eventually kill me”*

(Lucas).

*“You’re trapped in something that doesn’t belong to you… an imposter in your own body”*

(Martin).

Furthermore, Neil was able to describe how expressing thoughts of gender incongruence could lead to negative perceptions from others, furthering internalised negative thoughts:

*“You know what gender you are and imagine if everyone told you that… you’re not that gender and you are a bad person for thinking that you are that gender and that makes you delusional and disgusting”*

(Neil).

Such responses suggest that with age the participants were better able to express how their gender incongruence negatively impacted their well-being. They described a disconnection from their body, viewing it as not belonging to them, instead as a cage they existed as prisoners inside, where expressing incongruence would lead to negative perceptions from others. Therefore, this supports the minority stress model which explains that such stresses can lead to negative self-appraisals, like negative feelings about their body which in turn results in an internalising of stigma, negative body image and thus feelings of internalised shame [54].

Getting Older: ‘Self-stigma and maladaptive coping strategies’

As the participants got older, they described continuing difficulties with their well-being and internalising negative thoughts:

*“I was ashamed of who I was… I didn’t want to be alive… I felt very alone”*

(Lucas).

*“It was (a photo) of a sink and there’s no mirror above the sink because I don’t like looking at myself”*

(Neil).

**Figure 2:**
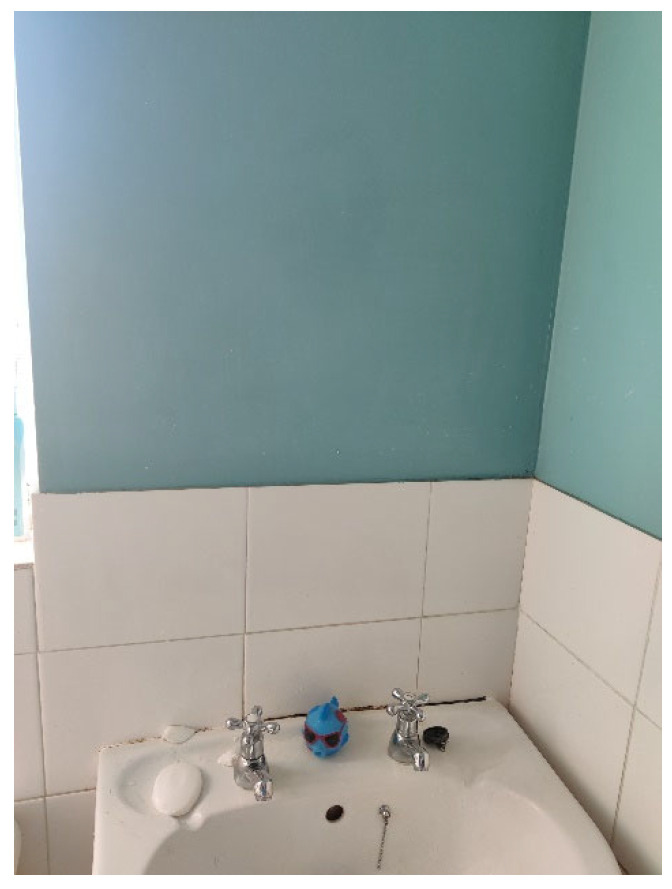
Neil’s photo of his bathroom without a mirror.

This suggests that as the participants approached early adulthood their well-being was further impacted as they continued to internalise feelings of shame and loneliness. Neil’s photo (See Figure 2) clearly illustrates the significant negative impact his own image has on his well-being. Being confronted with his reflection in the mirror is so distressing for him that he has removed the mirror from his bathroom to protect himself from having to accept his outward appearance and consequently as a means of coping with his negative feelings.

Furthermore, for some participants, they reached a point with their mental health where they turned to other unhealthy coping mechanisms as a means of dealing with their negative feelings:

*“My mental health… it kind of tipped over the edge and went down into… a chasm from which… I spent the next 50 years trying to recover”*

(Richard).

*“I did drink a lot… like particularly dysphoric days, I would just come home from work and I would just drink”*

(Ferb).

This suggests that for these participants such self-stigma is so distressing that they resort to maladaptive coping strategies, whether that be a refusal to face their outward appearance or drinking to cope with dysphoria. Therefore, such responses provide clear support for research demonstrating that gender dysphoria is a complicated paradigm of chronic stress involving the experience of distress and negative effects which impacts health throughout an individual’s lifespan [51]. Furthermore, for some, substance misuse is used as a coping mechanism to alleviate high stress levels, but this in turn leads to further negative impacts on their quality of life [55,56].

Reaching a Crisis Point: ‘A journey begins’

All of the participants then discussed a crisis point in their lives where they could no longer suppress their identities and had to live as their authentic selves:

*“There was a man in me trying to break through this massive barrier”*

(Ryan).

*“It wasn’t until my late 20’s where I realised I had two choices, either take my life or start this journey. I chose to start my journey and live with the consequences”*

(Lucas).

This suggests that the participants reached a critical point in their lives where they could no longer suppress their identities. Instead, they accepted their gender identities and started their transition journeys over the only alternative they thought they had, which would have been to take their lives. Furthermore, by reaching such a crisis point of accepting their true identities, this suggests a need for self-verification and a need to be seen by others in a way that matches their own self-image, thus demonstrating a fundamental desire for psychological coherence [57]. However, such acceptance of their identities resulted in significant threats to their mental health and well-being.

### 3.2. Well-Being Under Threat During Transition

The second theme centres around the significant threats to the participant’s well-being as they transition and is therefore made up of three sub-themes: family rejection, a transphobic society and navigating the NHS.

Family Rejection: ‘Conditional love’

Once the participants understood the need to start their transition, they explained that the first threat to their well-being was telling their family members. While some found support from their families, most of the participants described this as a very difficult time with the impact for some being so severe that their family rejected them entirely:

*“The majority of my family disowned me. My relationship with my parents is still rocky… they made my journey about themselves and how it affected them”*

(Lucas).

*“There’s been some distance from a lot of my family… who want to understand but can’t seem to wrap their heads around it. My Dad’s out of my life now, because of it”*

(Alan).

This suggests that for many of the participants, the accepting of their identities came with the sacrifice of their family relationships. The discovery of their parent’s conditional love and subsequent rejection may have caused significant distress, feelings of isolation and loss of belonging, thus further exacerbating their poor well-being. This provides further evidence to studies demonstrating that while familial support can be a protective factor against distress, transgender people experience difficulties in their family dynamics at all stages of their transition [58]. Furthermore, such family rejection can have a significantly negative impact on their mental health, leading to depression and suicidal thoughts in transgender adolescents [59,60].

A Transphobic Society: ‘A battle to exist’

The participants also discussed how the opinions and behaviours of society online and in-person threatened their well-being:

*“There’s so much transphobia in society… it just feels like a battle to stay alive”*

(Neil).

*“People are so hateful and we seem to be public enemy number one and the news is terrifying. It’s like people don’t want us to exist. They make flippant comments online… express opinions that literally kill people”*

(Lucas).

Subsequently, the participants felt a need to conceal their identity to avoid potential rejection and fit in with those around them, but such concealment further impacted their well-being:

*“I buried all my masculinity… I had to bury it so deeply to survive and prove to the world that I was normal… at great cost to me… in terms of my ability to function… and my career”*

(Ryan).

This suggests that due to such negative interactions, the participants felt they had to conceal their identity as a means of protecting themselves from rejection. Therefore, This once more supports the minority stress model where it is thought that such distal stressors like discrimination, prejudice and rejection result in transgender people developing proximal stress, which translates to internalised stigmatisation, identity concealment and rejection expectation [61,62]. Furthermore, this supports research demonstrating the amplificatory impact identity suppression has on emotional and psychological distress [52].

Navigating primary and secondary healthcare: ‘Gate-keepers of endless waiting’

All the participants described their experiences with the NHS gender transition process as a threat to their mental health and well-being, with the first challenge coming from their GPs having an apparent lack of knowledge regarding trans-related medical care:

*“… a lot of them (GPs) are either completely unsure what to do in general when they have a trans patient or they have their own opinions about what should be done… which isn’t necessarily in line with how the gender services think it should be done”*

(Alan).

*“When I first went to my GP about getting in contact with gender services, she’d never done it before. She was like how do I do that?”*

(Neil).

Such responses suggest that GPs were gatekeepers to the participants gaining access to gender-affirming medical care, yet due to their inexperience and lack of training they become a challenge instead of a support to the participants. Furthermore, once on the gender clinic waiting list, the most notable threat to the participant’s well-being was the length of time they had all been waiting for gender-affirming medical interventions on the NHS. The consequences of this were that 8 out of 11 participants paid to go private for their hormones and some surgeries:

*“I’ve been on the NHS waiting list for 10 years so far… and I’m looking at what could be up to a decade more… Being on the NHS waiting lists has ruined my life”*

(Lucas).

*“It’s definitely distressing… there’s no communication… you don’t get updates on how long the list is looking… you don’t hear anything until maybe a month before somebody wants to have a meeting with you”*

(Alan).

Additionally, Neil explained that he bought a binder as a temporary solution while waiting on surgery, but he has now been waiting on surgery for so long it has worn out.

*“My first binder… I thought I wouldn’t have to use it that long… but as you can see from the picture it’s already falling apart”*

(Neil).

**Figure 3:**
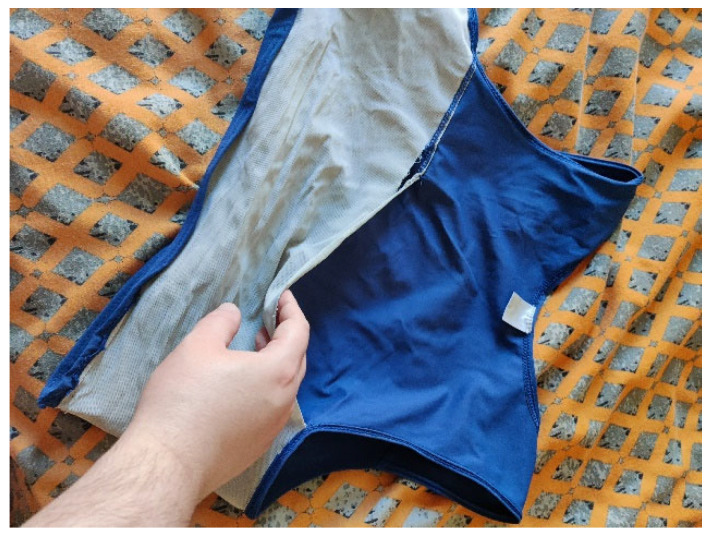
Neil’s photo of his binder.

This suggests that the inexperience of GPs, significant waiting times and a lack of communication from the NHS results in these participants not only facing challenges in getting on waiting lists, but they are then left on these waiting lists for multiple years, which has a negative impact on their well-being. Neil’s photo (See Figure 3) illustrates how these participants rely on what is supposed to be a short-term solution to aid their well-being, but instead they are relying on such solutions for so long that they are falling apart at the seams, which also serves as a physical representation of the state of their well-being when they are left on such long waiting lists. Furthermore, as medical intervention for these participants is the only means of escape from the bodies they are trapped in, participants are left living with uncertainty and being unable to progress with their lives, which subsequently has a negative impact on their mental health and well-being. This resonates with other research which reports that the two major obstacles for access to GICs are the knowledge gaps of medical professionals and long waiting lists, which are reported to be longer than any other specialist service in the UK [63]. Therefore, the cumulative effects of such difficulties in accessing services have resulted in an increase in poor mental health outcomes for transgender people and, thus, the need for transgender people to seek other sources for protecting their mental health and well-being [64].

### 3.3. Salutogenic Pathways to Positive Well-Being

The third theme highlights the key components which are fundamental in the protection and enhancement of the participants’ well-being, what Antonovsky [36] has called ‘salutory assets’. This theme is made up of four sub-themes that represent the most commonly identified resources: unwavering friendships, support of TGE peers, finding solace in nature and access to gender-affirming healthcare:

Unwavering Friendships: ‘Experiencing the unconditional’

With serious threats to their well-being and consequently being unable to find support from their families or the NHS, the participants described the ways in which they were able to protect their well-being, one of which was the unconditional support and acceptance of their friends:

*“… my friends, it didn’t affect my relationship with them really at all. Most of my friends I already knew and were supportive.”*

(Frank).

*“… I was really grumpy… (my friend)… posted it (a tea bag) through… I decided to pin it up… that is the pride and joy in it…”*

(Ferb).

**Figure 4:**
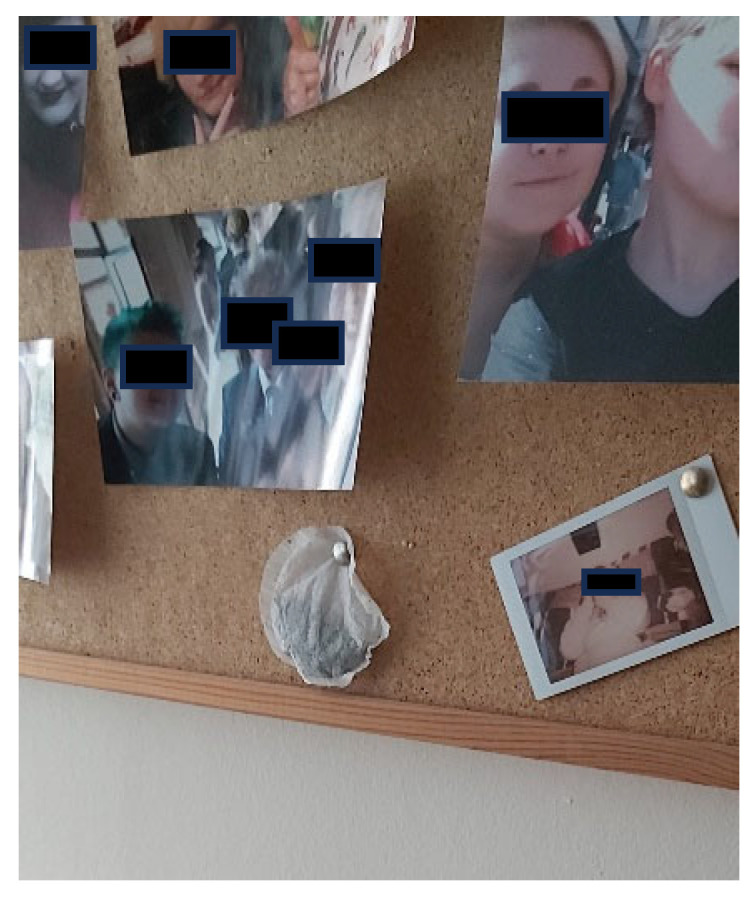
Ferb’s photograph of his friendship board.

This suggests that the participants rely heavily on the support and acceptance of their friend groups and that such unwavering friendships provide them with the much-needed support that was often lost from their family members. These friendships clearly act as a protective barrier from the often-negative experiences the participants have with wider society, giving them a sense of connection, continuity and belonging. This is illustrated nicely in Ferb’s photo (See Figure 4) where he explained telling his friend he was having a bad day during the COVID lockdowns of 2020. Within minutes his friend posted a tea bag through his door. Ferb saved the tea bag for two years before using it proudly displayed the tea bag as a symbol of his ‘*powerful friendship*’ on his friend board, clearly demonstrating how a small act of kindness, something as small as a tea bag was a powerful representation and reminder of friendship and support.

*“I have a lovely support system around me… it’s a very, lucky and blessed way of living that I’ve got… If I’m having a time where maybe I need another person around or something I can just drop a message in everybody’s like… not that far away. Just half a minute walk across the field to come and pop over”*

(Alan)

**Figure 5:**
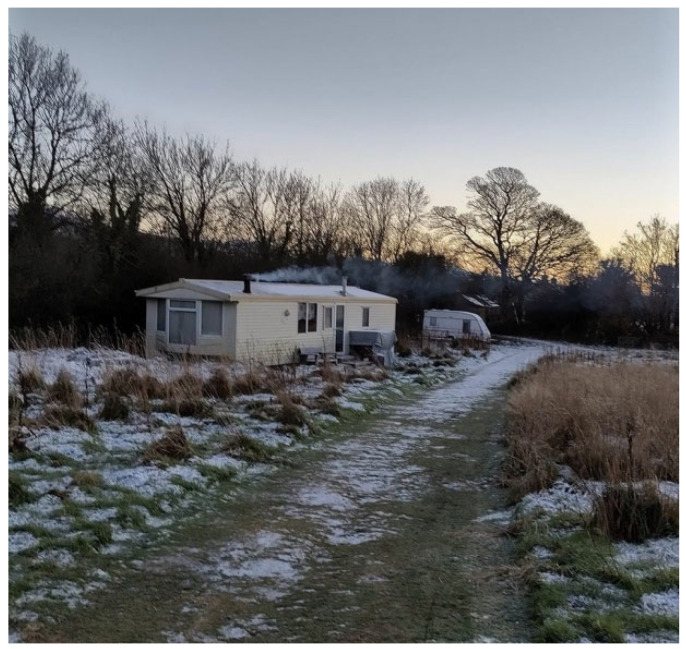
Alan’s photo of his caravan.

Alan, lives in a co-operative of fourteen people and pictured his caravan home (see Figure 5). He was able to count on the proximal care and support of his local friends and co-workers.

The availability and accessibility of trans-affirmative friends was of vital importance to most of our participants, especially when experiencing episodes of low mood and other negative emotions. Thus, such responses provide support for research which explains that transgender people may seek social support due to their stigmatised identities and that this pursuit of social support can have a positive effect on the emotional modulation of individuals with stigmas that are concealable [65,66]. Therefore, such social support is thought to be a pivotal protective factor for transgender people’s mental health and well-being and can have a positive impact on the ease of their transition process [67].

The Trans Community: ‘Support and solidarity’

The participants went on to discuss that along with their friends, they found significant support and solitude in members of the trans community:

*“… It gives you like a community of people with experiences like yours”*

(Ferb).

*“I need to draw on some solidarity with other trans people, support networks and this is how to survive”*

(Ryan).

Such responses suggest that the support of other transgender people who were going through the same experiences was essential to the participants. This was a key element for the participants surviving their transition journey as it appeared to give them a sense of comfort, belonging, solidarity and safety that they had been unable to find in the places mentioned previously. Thus, along with a fulfilment of such basic needs as a sense of belonging, the trans community clearly act as a protective barrier from those in the wider society who do not have an understanding of this group’s experiences. This supports research that has found that while a lack of peer support has been associated with depression in transgender people, the presence of support from others whose gender ‘falls outside of the norm’ gives them comfort, reduces the challenges of stigmatisation and acts as a buffer against the effects of minority stress [68,69], thus having a positive effect on their mental health [70].

Solace in Nature: ‘Connecting and belonging to something bigger’

Most participants believed that a connection with nature was important for their well-being and several other participants took photographs of their favourite scenes near where they lived:

*“It can be a nice thing where you’re feeling stressed or dysphoric… you just get outside… and sit amongst nature and clear your head… just connecting to something more tangible”*

(Alan)

*“the river… that’s my favourite place to go to, to just sort of relax and really enjoy nature as I find nature really sort of soothing and relaxing and it’s beneficial for mental health. OK. So that’s certainly one of my top definitely in my top things that I couldn’t, you know live without really getting outdoors and nature”*

(Richard)

**Figure 6:**
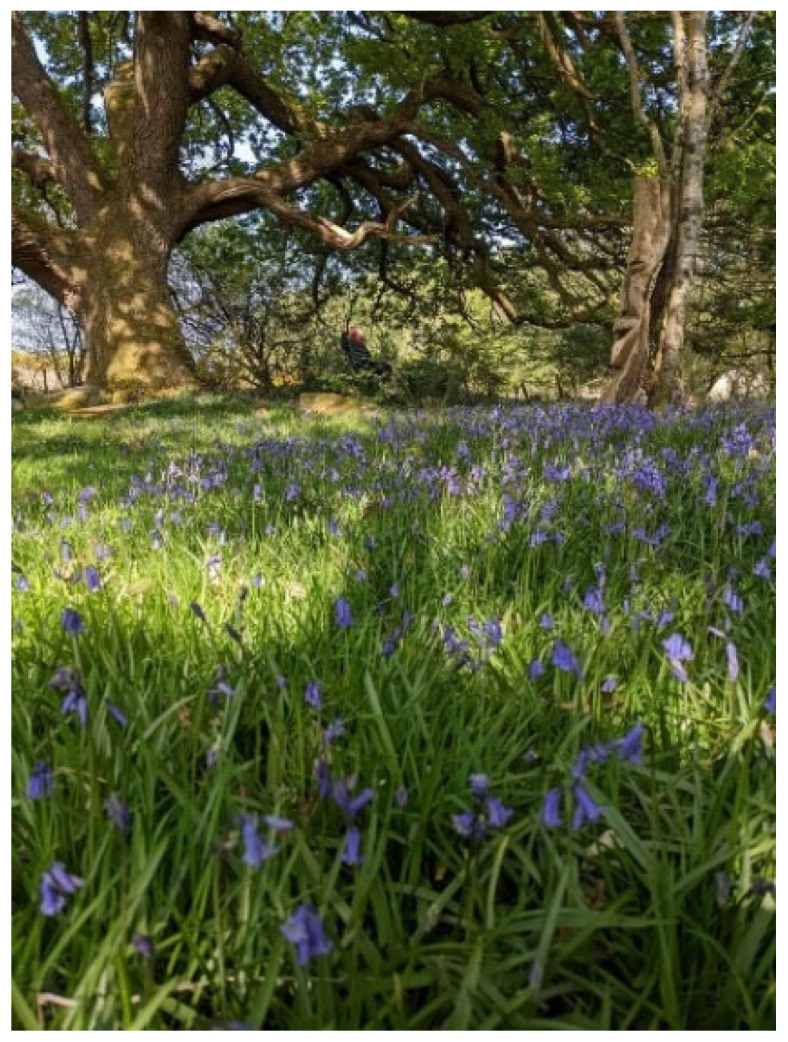
Alan’s photo of nature.

**Figure 7:**
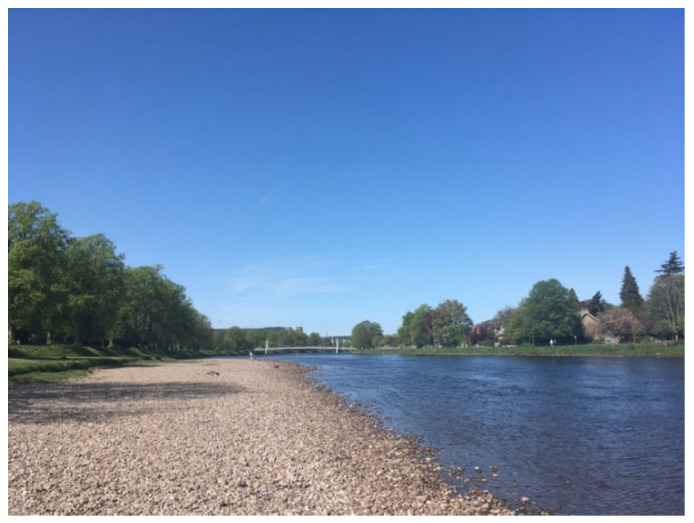
Richard’s photos of nature.

Such responses suggest that these participants view nature as a key salutogenic resource for their well-being, as clearly illustrated by Alan and Richard’s photo’s (See Figure 6 and Figure 7). Nature appears to give them a connection with something bigger than themselves, which suggests that as they search for belongingness in different aspects of their lives, nature may be a source of fulfilling their basic desire for belonging. This is echoed in other studies which have found that engagement in nature serves as a protective factor for psychological well-being, while nature connectedness is associated with a reduction in mental health symptoms, personal growth, purpose in life and positive effect [71].

**Figure 8:**
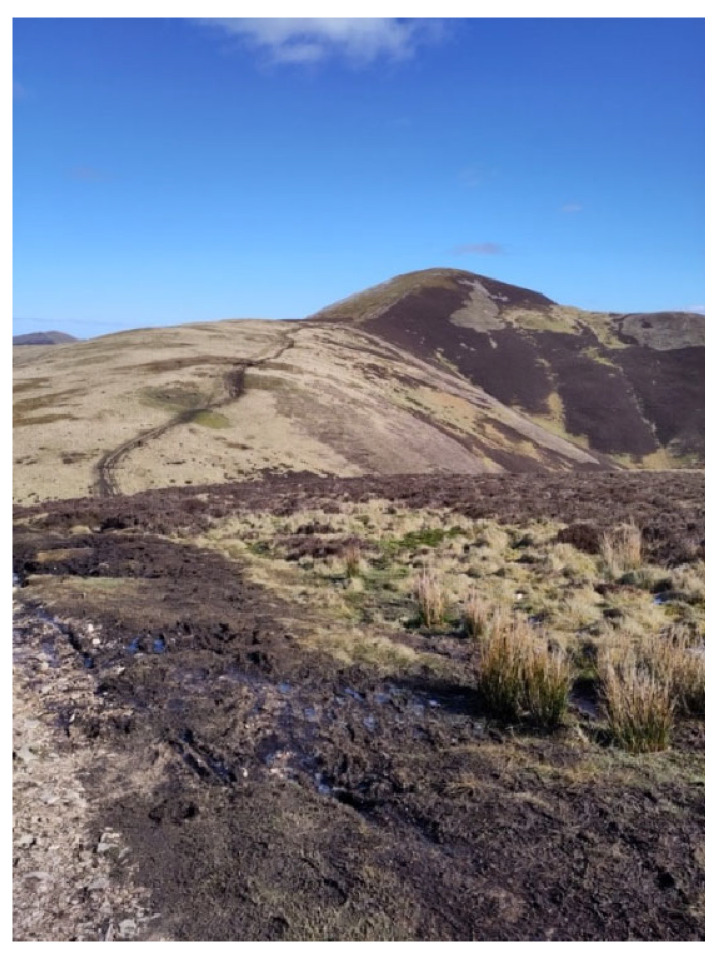
Ryan’s favourite hill-top.

Having said that, some participants alluded to a more complex relationship between nature-connectedness and well-being. Ryan took a photo of the Pentland Hills in Sussex, England (see Figure 8) and described his feelings when walking there as follows:

*“Deep communing, I suppose, with nature, which is, I mean, I’m not religious, but I think that’s my equivalent, really, of a spiritual life is to just feel that with the natural world.”*

However, he also alluded to a profound sense of sadness at reminders of reduced biodiversity and general environmental decline as well as noting the fleeting nature of the positive emotions experienced:

*“Sometimes it’s painful because I look around me and I love nature and I love bird watching, and I look at the birds and I just think there are fewer of them now… There are some birds I used to see a lot of that. I hardly see. So it’s painful. So I I don’t you know…, I have moments of joy, euphoria, even when I’m on a hilltop or in a wood and watching a tree creeper or, you know, long tailed tits or some other favourite birds. And I’m just. I can feel almost ecstatic. But it doesn’t last, you know”*

(Ryan)

Both psychologists and practitioners promote nature-connectedness through social prescribing. Without under-estimating the potential power of eco-therapies, we need to be vigilant that in a context of climate change and environmental damage we are aware of a sense of sadness that may accompany extended forays into the natural world.

Gender-affirming Healthcare: ‘A journey towards completeness’

The participants went on to discuss arguably the most profound impact on their well-being, which was having access to gender-affirming healthcare and living in a transformed body:

*“I can have a shower and I don’t have any disgust anymore… it’s literally life-saving surgery”*

(Martin).

*“I could definitely see… the improvement in the mental health that testosterone was giving me… I was able to reconnect with my own body”*

(Richard).

Such comments suggest that having access to surgery and hormones was life-saving for these participants. Before having access, they expressed feelings of disconnection and disgust with their bodies, yet surgery and hormones has given them a means to be happy with and reconnect with their bodies. Additionally, some participants further discussed the importance of their hormones and the feelings of completeness it gave them:

*“That’s a bit of a pun, that’s my tea table… I’ve also got my testosterone there… that is a very important thing… for my well-being”*

(Frank).

*“Hormones saved me. I wouldn’t be here without them or my surgeries… Every time I have had a surgery… I see myself as becoming or as if another piece of my jigsaw is complete”*

(Lucas).

**Figure 9:**
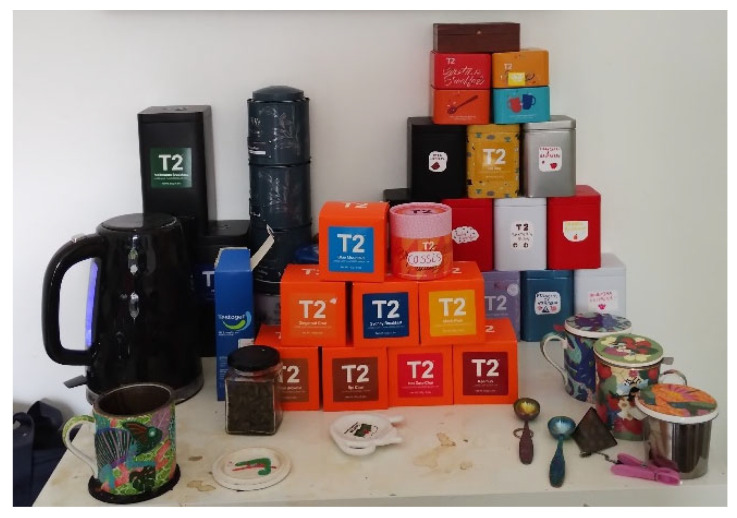
Frank’s photo of his ‘T’ table.

These responses suggest that the physical changes that surgery and hormones allow these participants provides them with a way of reconciling their external presentation with their internal identities, the importance of which is illustrated in Frank’s playful photo of his ‘T’ table (See Figure 9). These findings are echoed in other studies which have demonstrated that access to gender-affirming healthcare was essential in the elevation of distress in transgender people, reducing the symptoms of depression and anxiety, while improving self-esteem [72,73]. This reduction in depression and anxiety symptoms is believed to be due to individuals being happier with their body changes and thus experiencing fewer social psychological problems, which then results in an improvement in self-confidence, self-esteem and a better quality of life [72,74].

## 4. General Discussion

The findings of this study highlight the experiences of British transmen seeking medical intervention around their gender identity and the key ways in which they protect and cultivate their well-being as they navigate their transition. The participants reported feelings of discomfort and being different from a very young age. Such feelings were amplified during puberty when thoughts of suicide, depression and self-harm typically began. As they aged the participants reported continuing difficulties with their mental health and well-being which led to a pivotal point in their lives where they chose to begin their transition journeys, (typically with some familial opposition). These findings provide further evidence to research which had found such feelings of confusion and of being different were characteristic of young TGE people, with feelings of being out of place, depression and self-injurious behaviours amplifying during puberty and translating into further negative self-appraisals and thus supporting the minority stress model [49,54] and recent work on TGE identity development [3,37].

Moreover, reaching a crisis point of accepting their authentic identities supported research which demonstrates an individual’s need for self-verification and to be seen by others cohesively [57]. However, such self-acceptance resulted in threats to the participant’s well-being which were identified as rejection from family and members of their communities, difficulties accessing gender-affirming care from GPs and being left on significantly long NHS waiting lists. A lack of support from their family and medical professionals, along with feeling alienated from society, resulted in the continuing of internalised stigma, further supporting the minority stress model [61,62]. Therefore, the participants had to look to other sources for defending their well-being. They found this in unconditional friendships which provided support, acceptance and protection and with peers from the TGE community where they found a sense of belonging, comfort and solidarity. Spending time in nature gave them a sense of peace and connection with something bigger than themselves whilst, most notably, in the accessing of gender-affirming healthcare which allowed them to escape the bodies they felt they had been imprisoned within from a young age. Gender-affirming healthcare gave them the opportunity to reconcile their external presentation with their internal identities, alleviating symptoms of poor mental health and distress and, as shown in other studies, ultimately improving their quality of life and psychological well-being [73].

Participants reported finding the photographic component of the research enjoyable and accessible. We acknowledge that we used this component in a modest manner, partly because of concerns that requesting a more substantial contribution might appear overly burdensome. In future studies would aim to expand this component—potentially using more of a PhotoVoice paradigm where participants share their stories and photos in group discussions [75] and developing the action research potential of this methodology further. Cosgrove’s team argues that PhotoVoice group discussions can be especially empowering for TGE individuals promoting ‘self-expression, peer-connectedness, gender affirmation, and increased self-understanding’ [76].

Despite the depth of data provided, the study had a number of limitations. All participants recruited were white and we were unable to capture more diverse accounts. In future we would aim to work with trans organisations specifically for non-white people as this would allow for the involvement of transgender people from other ethnic backgrounds, which would hopefully lead to deeper insights into the intersections of faith and culture. All of our participants were attending social and/or support groups in Scotland or England, which also represents a rather skewed representation of the TGE community. Resource constraints also meant that participants were only interviewed once when ideally a more longitudinal approach would have been valuable. We set an inclusion criterion of accessing (or aiming to access medical intervention) and this therefore excluded many TGE individuals, especially transmen who were socially rather than medically transitioning and individuals born as women exploring more fluid or flexible forms of masculinity. Almost all our participants aligned with a “trapped in the wrong body” narrative and framed much of their accounts around this central idea whereas many trans* scholars and activists have rejected these constructions and explanations of gender dysphoria [77,78,79]. These aspects are important not only to better represent the diversity of the TGE community but also because the emerging literature suggests that experiences of prejudice and stigma may be different [80]. Whilst the findings of the study offer insights to the wider TGE community, especially where captured in two data media, the lack of heterogeneity in our sample does therefore arguably restrict the potential transferability of the findings and therefore some of their applications.

There remains a clear need to provide effective support systems to TGE individuals that take a strengths-based approach [81,82] and that foster peer community belonging [10]. Beyond extending opportunities for additional research, psychologists can support the well-being of transmen and other TGE-identifying people in multiple ways including through co-creating tailored anti-transphobic policies and developing stigma reduction interventions in organisations like schools, universities, hospitals and workplaces. Hughto and colleagues [83] developed a comprehensive socio-ecological model to inform stigma reduction interventions and in recent years various projects have tried to develop a range of interventions using positive emotions like compassion and empathy to try and increase acceptance and reduce prejudice [84]. We also need to develop further and deeper understandings of salutogenic resources (including and beyond those identified in this paper) that bring joy, contentment and connection to the lives of TGE people and communities [11,85] and consider the intersectionalities of non-white TGE individuals experiencing multiple stigmas of racism and transphobia [86]. Thorough understanding and recognition of the cultural and community contexts in which interventions are embedded is essential [87] and in particular dynamics within LGBT+ communities merit investigation and potentially intervention as these are not reliably positive environments for TGE individuals [10]. Whilst youth-based interventions are of critical importance, researchers, practitioners and policy-makers also need to be sensitized to the sometimes rather different needs of older members of the TGE community. Further research and training are also required for healthcare professionals, in both primary and specialised secondary care contexts, to help understand their perspectives of clinical and counselling encounters and enhance their knowledge and skills, as many report challenges in these areas [88,89] and would benefit from more examples of good practice.

Finally, it needs to be noted that after we completed data collection and analysis, in 2025, the British Supreme Court ruled that in a legal context the terms ‘man’ and ‘woman’ refer to biological sex and not certificated sex as had previously often been interpreted [90]. This ruling, generally rejected by TGE individuals, allies and activists, may have a very significant further influence on the healthcare of TGE individuals, the rights they hold under anti-discrimination laws and the ways that they are discussed in British society, and this development will be highly influential in research, training and healthcare practice going forward [90,91].

## 5. Conclusions

This study has provided contemporary, in-depth data to the typically understudied community of transmen in a British context. The findings presented in the first two themes confirm both the very harmful effects of individual and systemic prejudice on transmen. The final theme provides evidence of both the individual resilience of transmen and some of the salutogenic factors that they commonly utilise to support their well-being. These findings can go some way to understanding the needs of this community as well as informing more gender-affirmative healthcare and support for transmen in both clinical and community contexts.

## Figures and Tables

**Table 1:** Participant demographic information.

Pseudonym	Age	Gender Identity	Country of Residence
Tom	38	Man	England
Ryan	68	Transman	Scotland
Martin	32	Male	England
Richard	57	Male	Scotland
Louis	25	Transman/agender	Scotland
Curtis	19	Trans/male	England
Alan	22	Transmasculine	Wales
Neil	36	Transman/Demiguy	England
Ferb	23	Transman	England
Frank	23	Non-binary transman	England
Lucas	39	He	Scotland

**Figure A1:**
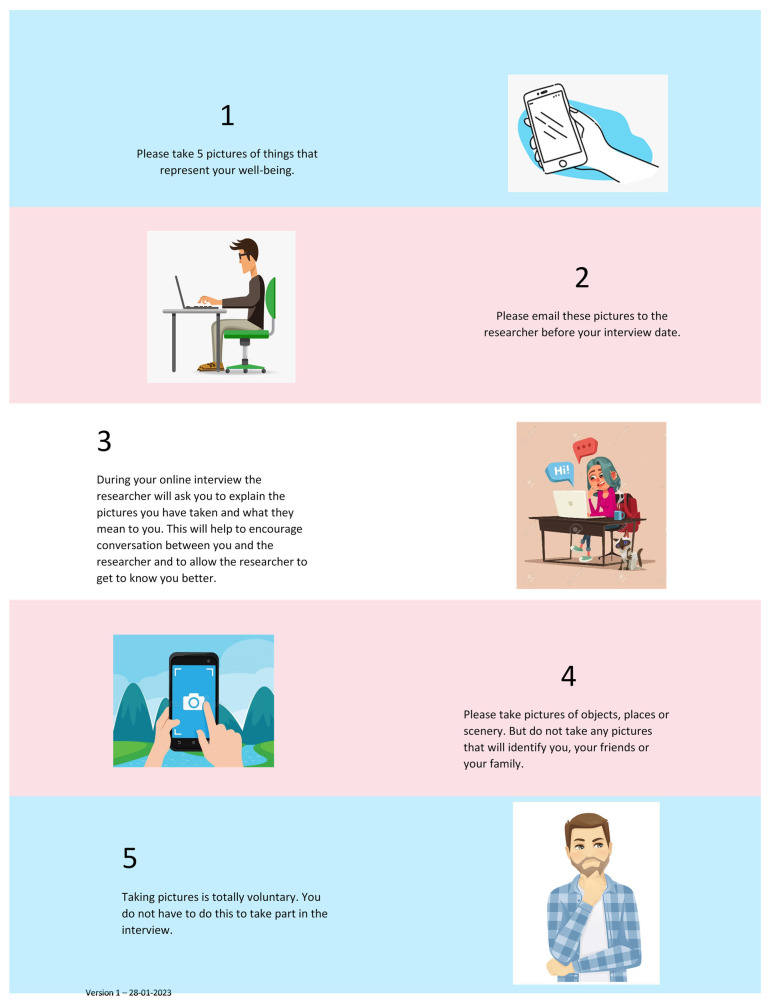
Photography Instruction Sheet.

## Data Availability

The datasets presented in this article are not readily available because of restrictions in consent from participants for wider distribution. Requests to access the datasets should be directed to the corresponding author.

## Appendix A. Interview Schedule

Would you like to tell me about a few of the pictures you took this week?

(1) What age were you when you first felt that you were Transgender/gender expansive? What was that like?
(2) How did these thoughts/feelings affect your well-being while you were growing up?
(3) How did telling your parents/friends/significant other affect your relationships?
(4) You do not have to tell me any details. But, how long have you been on/were on waiting lists for NHS medical intervention?
(5) How did being on these waiting lists affect your well-being?
(6) What has been your experience of any medical interventions you have had so far?
(7) How has this affected your mental health and well-being?
(8) Thinking of before you started to transition, how would you describe your well-being?
(9) Thinking of after you started to transition, how would you describe your well-being?
(10) If someone was to ask you what it is like to be Transgender, how would you describe this?
(11) What helps you maintain your well-being as you transition?
(12) Thinking of the future, what are your goals/what are you looking forward to?
(13) Is there anything important that you would like to add that you have not had the chance?
